# 3,5,6,7,8,3′,4′-Heptamethoxyflavone, a Citrus Polymethoxylated Flavone, Attenuates Inflammation in the Mouse Hippocampus

**DOI:** 10.3390/brainsci5020118

**Published:** 2015-04-15

**Authors:** Satoshi Okuyama, Kazuhiro Miyoshi, Yuichi Tsumura, Yoshiaki Amakura, Morio Yoshimura, Takashi Yoshida, Mitsunari Nakajima, Yoshiko Furukawa

**Affiliations:** 1 Department of Pharmaceutical Pharmacology, College of Pharmaceutical Sciences, Matsuyama University, 4-2 Bunkyo-cho, Matsuyama, Ehime 790-8578, Japan; E-Mails: abbarrl.abbarrl@gmail.com (K.M.); t_u1infiniy@yahoo.co.jp (Y.T.); mnakajim@cc.matsuyama-u.ac.jp (M.N.); furukawa@cc.matsuyama-u.ac.jp (Y.F.); 2 Department of Pharmacognosy, College of Pharmaceutical Sciences, Matsuyama University, 4-2 Bunkyo-cho, Matsuyama, Ehime 790-8578, Japan; E-Mails: amakura@cc.matsuyama-u.ac.jp (Y.A.); myoshimu@cc.matsuyama-u.ac.jp (M.Y.); xp769b@bma.biglobe.ne.jp (T.Y.)

**Keywords:** 3,5,6,7,8,3′,4′-heptamethoxyflavone, anti-inflammation, hippocampus, microglia, IL-1β

## Abstract

Citrus polymethoxylated flavones (PMFs) have recently been shown to suppress inflammation in peripheral tissues. In the present study, we investigated the effects of 3,5,6,7,8,3′,4′-heptamethoxyflavone (HMF), one of the PMFs, on inflammation in the brain *in vivo* using mice injected intrahippocampally with lipopolysaccharide (LPS). We demonstrated that subcutaneously injected HMF suppressed: (1) LPS-induced losses in body weight; (2) LPS-induced microglial activation in the hippocampus; and (3) LPS-induced interleukin-1β mRNA expression in the hippocampus. These results suggest that HMF has the ability to reduce neuroinflammation in the brain.

## 1. Introduction

Inflammation is a normal physiological response to infection/injury; however, its over-activation sometimes becomes toxic. Recent studies have shown that the pathophysiology of numerous diseases/lesions in the central nervous system (CNS) are also associated with neuroinflammatory responses, such as ischemic stroke [1], traumatic brain injury [2] and various neurodegenerative disorders, including Alzheimer’s disease (AD) and Parkinson disease (PD) [3]. Studies on treatments with inflammatory modulators, such as non-steroidal anti-inflammatory drugs (NSAIDs) for animal models of AD [4] or ischemic brain injury [5], began in the early 1990s. In the last ten years, natural compounds from traditional herbs have been receiving attention for the treatment of ischemia [6], because they exhibit multiple actions that may prevent neurodegenerative processes involving various/complex mechanisms.

**Figure 1:**
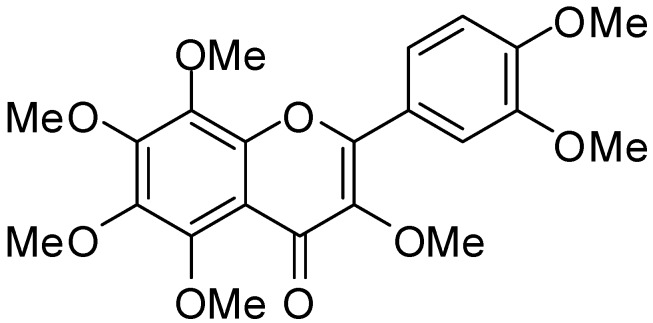
Structure of 3,5,6,7,8,3′,4′-heptamethoxyflavone (HMF).

Among natural materials, citrus fruits are some of the most popular fruits worldwide and are known to be a rich source of bioactive compounds, including flavonoids, coumarins, limonoids and carotenoids. Particularly in flavonoids, polymethoxylated flavones (PMFs) and hydroxylated polymethoxyflavones are sure to be the focus of recent attention, because of their broad spectrum of biological activities [7]. PMFs were shown to have anti-inflammatory effects in peripheral tissues, for example: (1) 3,5,6,7,8,3′,4′-heptamethoxyflavone (HMF; Figure 1) exhibited anti-inflammatory effects on isolated human monocytes that were mediated by the inhibition of phosphodiesterase (PDE)-4 and cytokine production [8]; (2) 5,6,7,8,3′,4′-hexamethoxyflavone (nobiletin; NBT) attenuated lipopolysaccharide (LPS)-induced inflammatory factors in human synovial fibroblasts and mouse macrophages [9]; (3) 5-hydroxy-3,6,7,8,3′,4′-hexamethoxyflavone inhibited 12-*O*-tetradecanoylphorbol 13-acetate (TPA)-induced skin inflammation and tumor promotion in mice [10]; (4) 5,6,7,8,3′,4′,-hexamethoxy-3-hydroxyflavone (natsudaidain) inhibited the expression of tumor necrosis factor (TNF)-α and cyclooxygenase (COX)-2 in Ca^2+^ ionophore-stimulated rat basophilic leukemia cells (A23187-stimulated RBL-2H3 cells) [11]; and (5) 5,7,4′-trihydroxy-6,8,3′-trimethoxyflavone (sudachitin) suppressed the expression of TNF-α and the production of nitric oxide (NO) in LPS-stimulated RAW264 cells, a macrophage-derived mouse cell line [12]. Recent *in vitro* studies suggested that HMF may have also anti-inflammatory effects in CNS-related cells; that is, HMF has been shown to inhibit the production of NO and inducible nitric oxide synthase (iNOS) in cultured astrocytes, which are involved in supporting neurons in the CNS [13]. In our *in vivo* study, HMF suppressed the microglia activation in the hippocampus following ischemia and protected from neuronal cell death [14]. Therefore, the aim of the present study was to investigate whether HMF exhibited anti-inflammatory activity in the LPS-injected brain.

## 2. Experimental Section

### 2.1. Animals

Eight-week-old male C57BL/6 mice were purchased from Japan SLC (Hamamatsu, Japan). Mice in all groups were kept at 23 ± 1 °C and on a 12-h light/dark cycle (light on 8:00~20:00). Tap water and food were freely available during the experimental period. All animal experiments were carried out in accordance with the Declaration of Helsinki and the Guidelines for Animal Experimentation prepared by the Animal Care and Use Committee of Matsuyama University (approved in 2 September 2009, Protocol No. 09-002).

### 2.2. HMF Treatment

HMF was prepared from commercial orange oil (Wako, Osaka, Japan) as described previously [15], and dissolved in dimethyl sulfoxide (DMSO)/polyethylene glycol (PEG) 300 (1:1) solution at the concentration of 20 mg/mL. Mice were subcutaneously (*s.c.*) administered HMF to achieve 100 mg/kg/day (LPS + HMF100 group; *n* = 9), because this dose of HMF has been shown to be more than sufficient to exert its neuroprotective effects in ischemic brain [14,16]. Mice in another two groups (CON; control group, *n* = 6; and LPS group, *n* = 11) were treated with vehicle (DMSO/PEG300). A volume of HMF solution or vehicle was 0.125 mL per 25 g mouse. The *s.c.* administration of these samples was continued once a day for ten days (Day 1~Day 10).

### 2.3. LPS Intrahippocampal Challenge

LPS (from *Salmonella enterica* serotype typhimurium) was purchased from Sigma-Aldrich (St. Louis, MO, USA) and dissolved with phosphate-buffered saline (PBS) at the concentration of 7.5 mg/mL. The surgery for the intrahippocampal administration of LPS was performed immediately after the injection of the samples (vehicle or HMF) on the eighth day (Day 8). After mice were immobilized in a stereotaxic device (Stoelting, Wood Dale, IL, USA) under pentobarbital anesthesia, a linear skin incision was made over the bregma, and a 1-mm burr hole was drilled in the skull 2.3-mm posterior and 1.8-mm lateral to the bregma on both sides using a hand-held driller. Two microliters of LPS solution (the dose of LPS was 15 μg) were injected 2.0 mm below the surface of the skull using a 10-μL Hamilton syringe for each hemisphere; therefore, 30 μg/4 μL of sample were injected for both hemispheres in mice.

Two days after the LPS injection (Day 10), mice were anesthetized and transcardially perfused with ice-cold PBS. Their brains were then removed, and one of the hemispheres of each brain was used for immunohistochemistry while the other hemisphere was used for RT-PCR.

### 2.4. Immunohistochemistry

Brains were postfixed as described in a previous study [16]. The frozen hemispheres were sagittally sectioned at 30 μm using a cryostat (CM3050S; Leica Microsystems, Heidelberger, Germany) for optical microscopy. The primary antibody and secondary antibody for optical microscopy were a rabbit polyclonal antibody against ionized calcium binding adaptor molecule 1 (IBA1, dilution 1:1000; Wako, Osaka, Japan) and EnVision-plus system-HRP labeled polymer (anti-rabbit; Dako, Glostrup, Denmark), respectively. Immunostained sections were visualized with DAB substrate (SK-4100; Vector Laboratories, Burlingame, CA, USA), and immunoreactive signals in the stratum radiatum and stratum lacunosum-moleculare of the hippocampus, which is the injection site, were captured using an optical microscope (CX21; Olympus, Tokyo, Japan). Three sections per mouse were quantified using the particle size measurement of Image J software (NIH, Bethesda, MD, USA).

### 2.5. RT-PCR Procedures

Total RNA from the hippocampal region of the mice was prepared by use of Isogen (Nippon Gene, Tokyo, Japan), composed of phenol and guanidine thiocyanate and then transcribed into cDNA, as described in a previous report [17]. The synthesized cDNA was amplified by PCR using pairs of primers for COX-2, iNOS, TNF-α, interleukin (IL)-1β and β-actin. The numbers of PCR cycles, specific annealing temperature and primer pairs are in Table 1. Reaction products were electrophoresed on 2% agarose gels containing ethidium bromide. The intensity was measured by using a FAS-Ш imaging system (TOYOBO, Osaka, Japan).

**Table 1:** Primer sequences and PCR condition.

Gene	Direction	Primer Sequence (5′ to 3′)	Cycles	Denaturation	Annealing	Elongation
COX-2	forward	5′-AAGGCCTCCATTGACCAG-3′	32	94 °C	56 °C	72 °C
	reverse	5′-TCTTACAGCTCAGTTGAACGC-3′				
iNOS	forward	5′-CCCTTCCGAAGTTTCTGGCAGCAGC-3′	35	94 °C	65 °C	72 °C
	reverse	5′-GGCTGTCAGAGAGCCTCGTGGCTTTGG-3′				
IL-1β	forward	5′-CTTGGGCTGTCCAGATGAGAGCAT-3′	33	94 °C	63 °C	72 °C
	reverse	5′-GAAGACACGGGTTCCATGGTGAAG-3′				
TNF-α	forward	5′-ATGAGCACAGAAAGCATGAT-3′	35	94 °C	50 °C	72 °C
	reverse	5′-TGACTTTCTCCTGGTATGA-3′				
β-Actin	forward	5′-CATGTTGAGACCTTCAACACCCC-3′	37	94 °C	60 °C	72 °C
	reverse	5′-GCCATCTCCTGCTCGAAGTCTAG-3′				

### 2.6. Statistical Analysis

Data for the individual groups are expressed as the means ± SEM. Data were analyzed by a one-factor ANOVA followed by Tukey’s multiple comparisons test (Prism 6; GraphPad Software, La Jolla, CA, USA). A value of *p* < 0.05 was considered to be significant.

## 3. Results

### 3.1. HMF Suppressed Body Weight Loss

**Figure 2:**
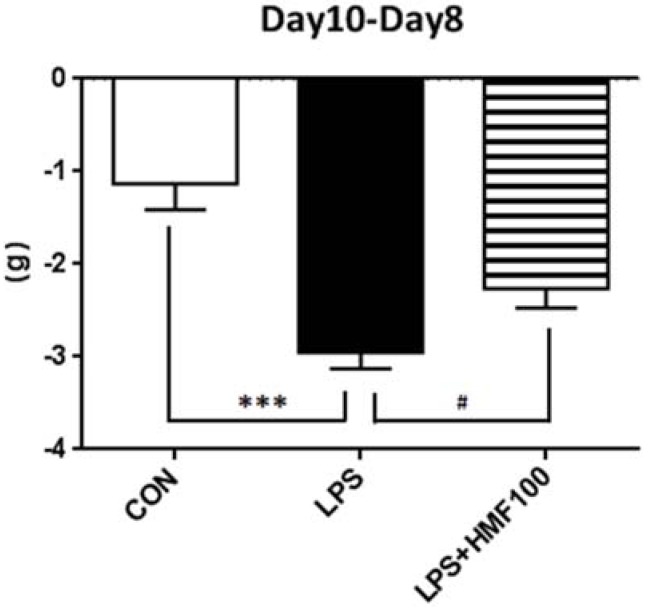
Effects of the subcutaneous administration of HMF on LPS-induced losses in body weight. Values are the means ± SEM. Symbols are significantly different for the following conditions: CON; control group *vs*. LPS, *** *p* < 0.001; LPS *vs*. LPS + HMF100, ^#^ *p* < 0.05.

In order to examine the effects of HMF on LPS-induced sickness responses, we assessed body weights two days after the injection of LPS before mice were sacrificed. As shown in Figure 2, significant body weight loss was noted in the LPS group (*** *p* < 0.001 *vs.* CON group), and the HMF treatment (100 mg/kg/day) significantly suppressed LPS-induced body weight loss (^#^ *p* < 0.05 *vs.* LPS group).

### 3.2. Effects of HMF on the LPS-Induced Activation of Microglia

**Figure 3:**
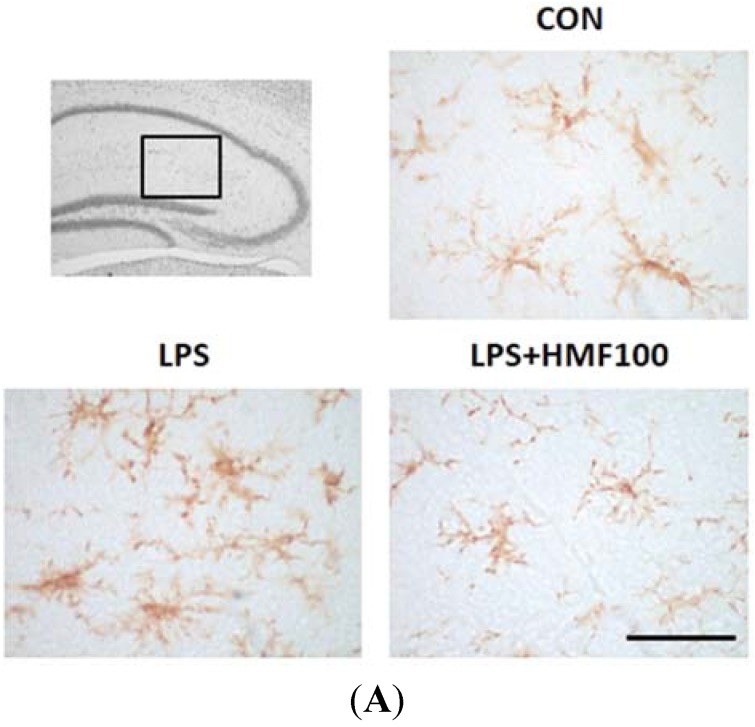

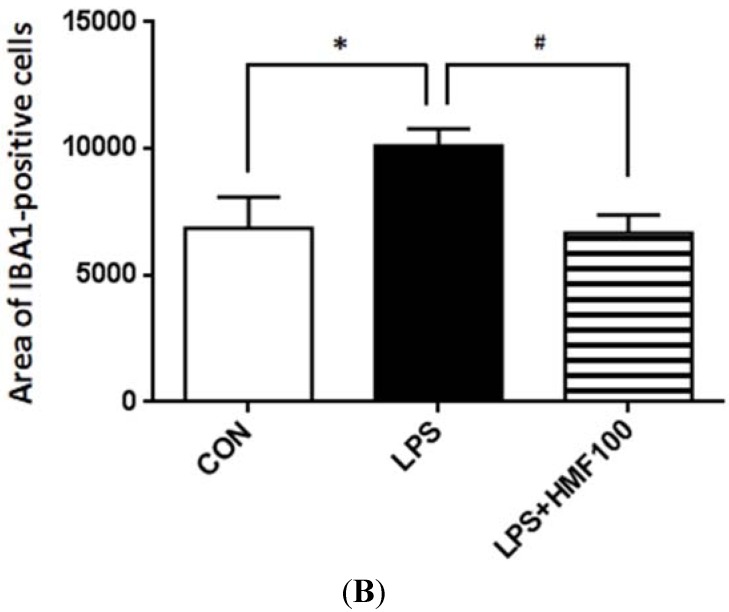
Effects of HMF on the expression of microglia (IBA1) in the mouse hippocampus. The location of the captured images and quantification are shown with squares in the figure. (**A**) Sagittal sections of the hippocampus two days after the LPS intrahippocampal injection stained with an anti-IBA1 antibody; CON, LPS, LPS + HMF100. The scale bar shows 50 µm; (**B**) Quantitative analysis using Image J software of the total areas (pixels) of IBA1-positive cells in the hippocampus. Values are the means ± SEM. Symbols are significantly different for the following conditions: CON *vs*. LPS, * *p* < 0.05; LPS *vs*. LPS + HMF100, ^#^ *p* < 0.05.

We investigated the effects of HMF on LPS-induced microglial overactivation. Microglial cells were immunostained with the IBA1 antibody. In all experimental groups, IBA1 immunoreactivity was detected in the stratum radiatum and stratum lacunosum-moleculare of the hippocampus that is near the LPS injection site (Figure 3A). IBA1-positive cells with the inactivated form (ramified microglia) were observed in the CON group. The morphology of IBA1-positive microglia was changed to “ameboid microglia” (the activated form) by the treatment with LPS, and the total area of these cells significantly increased (Figure 3B; * *p* < 0.05); on the other hand, HMF (100 mg/kg/day) treatment significantly suppressed the LPS-induced activation of microglia (Figure 3B; ^#^ *p* < 0.05).

### 3.3. Effect of HMF on the LPS-Induced Expression of Proinflammatory Mediator and Cytokines

**Figure 4:**
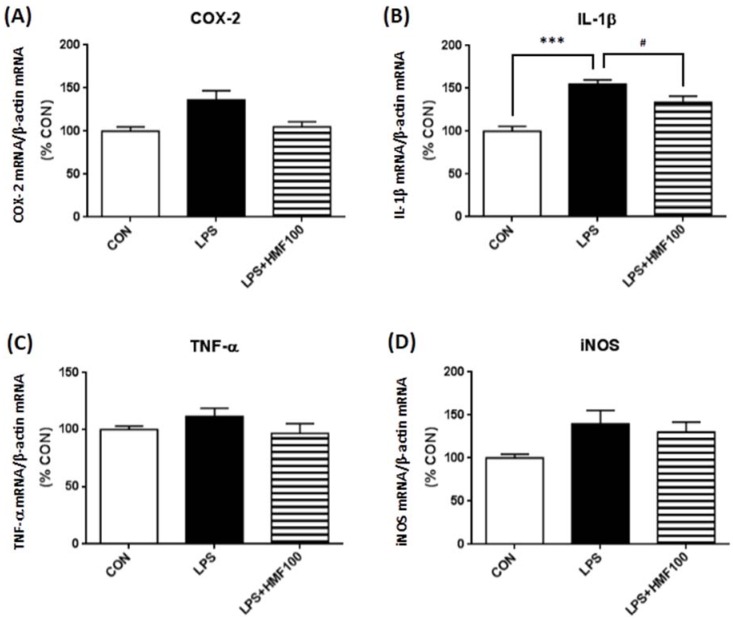
Effects of HMF on the expression of mRNAs for inflammatory markers in the hippocampus. Hippocampal tissues were prepared two days after LPS intrahippocampal injection, and RT-PCR analysis was performed with specific primers. Densitometric quantification of mRNAs of COX-2 (**A**), IL-1β (**B**), TNF-α (**C**) and iNOS (**D**) band intensities normalized by the β-actin mRNA band in the hippocampus. Values are the means ± SEM. Symbols are significantly different for the following conditions: CON *vs*. LPS, *** *p* < 0.001; LPS *vs*. LPS + HMF100, ^#^ *p* < 0.05.

As activated microglia are known to contribute to neurodegenerative processes by producing various cytotoxic molecules, including proinflammatory cytokines [18,19,20], we examined the effects of HMF on the LPS-induced gene expression of proinflammatory cytokines (IL-1β and TNF-α) and enzyme (iNOS and COX-2) with RT-PCR. Figure 4B shows that IL-1β mRNA expression in the LPS group was significantly higher than that of the CON group (*** *p* < 0.001), and HMF treatment suppressed its increase (^#^ *p* < 0.05). TNF-α mRNA and iNOS mRNA expression were not increased by LPS, and the treatment with HMF did not change its level. COX-2 mRNA expression in the LPS group was higher than that of the CON group, but not significant (*p* = 0.052), and this increment showed the tendency to suppress by the treatment of HMF at the concentration of 100 mg/kg/day (*p* = 0.056; Figure 4A).

## 4. Discussion

In the present study, we investigated whether HMF, one of the citrus PMFs, exhibited anti-inflammatory effects on the LPS-injected mouse brain. This is because previous findings indicated that the suppression of inflammation in the brain may be important for improving neurodegenerative pathologies and related brain functions. For example, in the ischemic brain, elements of the immune response participate in all stages of the ischemic cascade [21], and the inhibition of COX-2 is shown to prevent the delayed death of CA1 hippocampal neurons following global ischemia [22]. In the AD brain, the accumulation of amyloid β has been associated with immune responses [4], and anti-inflammatory drugs, such as NSAIDs, are shown to reduce the risk of AD [23].

Among the many animal models of neuroinflammation, we used mice injected intrahippocampally with LPS, a Gram-negative bacterial cell surface proteoglycan, in the present study. LPS binds to toll-like receptor (TLR) 4, and its expression in the brain has been detected in the meninges, ventricular ependyma, circumventricular organs, along the vasculature and in parenchymal microglia [24]. A previous study reported that the inflammatory response induced in the brain by an intrahippocampal injection of LPS was primarily mediated by microglia [25]. Microglia, which are responsible for protecting the CNS against various types of pathogenic factors under physiological conditions, are readily activated in response to injuries or immunological challenges [26]. In order to assess the anti-inflammatory effect of HMF, we evaluated its effect on the microglial activation by immunohistochemical analysis (Figure 3).

Microglial activation has been shown to activate more microglia, as well as a self-propelling progressive cycle of inflammation [27]. The over-activation of microglia and subsequently excessive production of inflammatory cytokines are known to be neurotoxic [26]; therefore, we then valuated the level of mRNA expression of inflammatory factors (TNF-α, IL-1β, iNOS and COX-2) by RT-PCR. RT-PCR showed that HMF significantly suppressed the LPS-induced expression of IL-1β mRNA in the hippocampus (Figure 4B). These data were consistent with the previous finding that the level of IL-1β mRNA in the hippocampus was still significantly higher after three days following LPS intracerebroventricular injection [28]. In contrast, neither TNF-α mRNA, iNOS mRNA nor COX-2 mRNA was changed by LPS, probably because the expression of these factors may have returned to control levels within several hours after the LPS stimulation [29,30]. In fact, LPS triggers proinflammatory cytokine production, with TNF-α induction first, followed by IL-1β [31,32] and in the brain [33,34]. Unfortunately, we could not detect the protein of these cytokines by Western blot analysis of hippocampus tissues. In the future experiment, we will have to analyze cytokine work at much shorter time points.

We have not yet investigated the mechanism responsible for the anti-inflammatory effects of HMF. Nuclear factor-kappa B (NFκB) and/or mitogen-activated protein kinase (MAPK; ERK1/2, JNK, p38) are known to be involved in the LPS-induced inflammatory response, and NBT was recently reported to suppress LPS-induced TNF-α, IL-1β and iNOS in BV2 microglia by inhibiting NFκB and phospho-MAPKs [35]. The inhibitors of PDE have also been shown to suppress the production of TNF-α [36]. HMF showed the ability to inhibit PDE4 activity and suppressed TNF-α production in human monocytes and mouse serum [8,37], and microglia express PDE4 [38]. These findings suggested that HMF might exhibit an anti-inflammatory ability by inhibiting NFκB and/or MAPK as well as NBT does.

In the present study, we also showed that the administration of HMF suppressed LPS-induced body weight loss (Figure 2). Previous studies demonstrated that intracerebroventricular injection of LPS or IL-1β induces sickness behavior, anorexia and body weight loss [39]. The balance of two pathways, the leptin (adipocyte-derived hormone)-PI3K-PDE pathway and the ghrelin (appetite-related hormone)-adenylate cyclase-cAMP-PKA system in the hypothalamus, were related to the feeding mechanisms [40]. As a main possibility, HMF inhibited inflammation in the brain and recovered food intake and body weight loss, and as other mechanisms, HMF might suppress inflammation-induced anorexia by ameliorating the balance of these two feeding systems, because: (1) rikkunshito, a traditional Japanese medicine to treat various gastrointestinal disorders, suppressed cisplatin-induced anorexia [41]; (2) rikkunshito ameliorated the aging-associated decrease in food intake by inhibiting PDE3 [40], and HMF, one of the important components of rikkunshito, exerted the inhibitory effect of PDE3; (3) rikkunshito altered the balance of two systems (the leptin-PI3K-PDE pathway and the ghrelin-adenylate cyclase-cAMP-PKA system) in the hypothalamus.

In the present study, HMF was administered by *s.c.* injection, as we previously showed that *s.c.* administration of HMF suppressed the ischemic-induced microglia activation and neuronal cell death in the hippocampus [14]. In order to use HMF as a therapeutic agent in the future, we will have to examine whether: (1) HMF can exert the anti-inflammatory effect after oral administration; (2) peripherally-administrated HMF can pass through into brain; and (3) HMF can suppress inflammation-induced neuronal cell death.

## 5. Conclusions

HMF had anti-inflammatory action in the brain and suppressed inflammation-induced body weight loss. HMF might be beneficial as a therapeutic/preventive compound for various neurological diseases in humans.
